# Psychopathological and Interactive-Relational Characteristics in Non-Suicidal Self-Injury Adolescent Outpatients

**DOI:** 10.3390/jcm11051218

**Published:** 2022-02-24

**Authors:** Marina Miscioscia, Caterina Angelico, Alessia Raffagnato, Michela Gatta

**Affiliations:** 1 Child and Adolescent Neuropsychiatric Unit, Department of Women’s and Children’s Health, University Hospital of Padua, 35128 Padova, Italy; caterina.angelico@gmail.com (C.A.); alessia.raffagnato@unipd.it (A.R.); michela.gatta@unipd.it (M.G.); 2 Department of Developmental and Social Psychology, University of Padua, 35131 Padova, Italy

**Keywords:** NSSI, non-suicidal self-injury, adolescence, Lausanne Trilogue Play, alexithymia, family interaction dynamics, developmental psychopathology

## Abstract

Non-suicidal self-injury (NSSI) is described as behaviors that directly and intentionally inflict damage to body tissue without suicidal intent and for reasons not linked to cultural expectations or norms. Literature has confirmed several “specific risk factors” related to NSSI behaviors; emotional reactivity, internalizing problems, alexithymia traits, and maladaptive family functioning can predispose an individual to intrapersonal and interpersonal vulnerabilities related to difficulties in regulating one’s own cognitive-emotional experience. The present study aims to analyze and define the psychopathological and family interactive-relational characteristics of adolescents with NSSI through a case-control study. Thirty-one patients with NSSI and thirty-one patients without NSSI paired for sex, age, and psychiatric diagnosis (ICD-10) were recruited in Padua among two Child Neuropsychiatry Units before the COVID-19 pandemic. Results show a higher prevalence of internalizing problems, alexithymia trait related to “difficulty identifying feelings”, and lower quality of family functioning related to inclusion of partners, child involvement, and child self-regulation. These results carry significant implications for the clinical management and therapeutic care of non-suicidal self-injury patients and further confirm the need for an in-depth investigation of internalizing problems, alexithymia, and quality of family interactions.

## 1. Introduction

Non-suicidal self-injury (NSSI) is described as “directly and intentionally inflicting damage to one’s own body tissue without clear suicidal intent and for reasons that are not consistent with cultural expectations or norms” [1]. Several reviews have identified a high prevalence of NSSI in adolescence [2]. A meta-analysis by Swannell et al. [3] has shown that the worldwide lifetime prevalence of NSSI in a community sample of adolescents is 17.2%; this rate significantly increases in clinical samples, with a prevalence of 40% or more among adolescents with psychiatric impairment [4]. The age of onset of NSSI is between 12 and 14 years [5,6]; a recent study by Voss et al. [7] confirms that females show a prevalence estimate more than twice as high than males. NSSI episodes in adolescence are often repeated, particularly among those who self-harm by cutting; more than 55% do so more than once [8] and may occur in single or multiple (≥4/year) episodes, revealing habitual and non-habitual patterns of self-injury [9].

Genetic factors, emotional reactivity, a history of abuse, and family functioning, can predispose an individual to the risk of intrapersonal and interpersonal vulnerabilities related to difficulties in regulating one’s own cognitive-emotional experience and to the risk of facing social problems [1]. In this context, NSSI acquires the function of regulating these experiences. Individuals may engage in NSSI due to “specific risk factors”, namely, as a result of social learning (exposure to others engaging in NSSI), as self-punishment, as a tool to communicate with others, because of its pragmatic functionality, because endorphin-induced pain analgesia, or as a result of implicit identification with NSSI [1].

A systematic review of literature, conducted between 1998 and 2016 by Cipriano et al. [10] highlighted a frequent association between NSSI and several psychiatric disorders, both internalizing and externalizing; among the main ones, mood disorders, anxiety disorders, post-traumatic stress disorder, feeding and eating disorders, substance use disorder, conduct disorder, intermittent explosive disorder, and borderline personality disorder are mentioned.

Alexithymia plays an important role in the association with the onset of NSSI due to emotional awareness difficulties [11,12]. Individuals who engage in NSSI show vulnerability in managing emotions on an individual and interpersonal level, which can be identified in their difficulty recognizing and communicating their own and other people’s feelings. Faced with emotional dysregulation and lacking the strategies to manage negative emotions [1,13], the use of one’s own body as a substitute for words prevails as a means of expressing one’s feelings [14]. Individuals with higher levels of alexithymic traits and poor emotion differentiation skills could, therefore, employ impulsive-aggressive behaviors mediated by emotion dysregulation [15].

Research on family functioning identified family relationships, attachment, maltreatment, socialization, scapegoating, cohesion [16,17,18], stressful or maladaptive family functioning, tense family climate or parental separation, family history of self-harm or suicidal behavior, psychopathology, alcohol or substance abuse in one of two parents [19] as significant NSSI-related risk factors.

In particular, dysfunctional family dynamics seem to be characterized by a lack of positive emotional exchange between family members, a hostile parenting style, lack of autonomy support, hyper controlling and critical, and interactive family dynamics were revealing triangulation difficulties [20,21]. In the domain of parent-child relationship, a study by Di Pierro et al. [22] found that an inadequate mother-child relationship correlated not only with the presence of non-suicidal self-injury but also with its severity, while the father-child relationship influences the intensity of the act itself. A recent longitudinal study [23] investigating the role of the parent-child relationship in the development of NSSI showed that a “dysfunctional” maternal attachment could entail a more significant risk of NSSI through an altered process of identity formation/synthesis. Therefore, such a relationship appears to activate a cascade of identity development and growth difficulties in the child. Some authors who investigated the consequences of NSSI on other family members found that self-harm activates what could be described as a vicious circle. The child’s self-harming behavior leads to emotions such as fear, shame, and confusion, which can affect the parent’s own behavior and, consequently, their relationship with the child, which may lead to an increased risk for NSSI [24]. Family functioning, quality of the parent-child relationship, and other parental factors can influence the onset of self-harming behaviors. At the same time, NSSI can, in turn, damage the parents’ ability to face the situation [25].

Lumley et al. [26] found a specific association between the different dimensions of child alexithymia and family functioning. In particular, the quality of family involvement is associated with difficulties in emotion identification; low family control and a lack of rules are related to externally oriented thinking. Parental problem-solving ability influences the child’s imagination and symbolic thought. During family exchanges, the adolescent’s communications regarding personal experiences and emotions are often ignored, trivialized, or punished, instead of being encouraged and supported [27].

The quality of family and social environment may be particularly important during the onset and the repetition of NSSI [28]; a recent study of Nemati et al. [29] found that low levels of family psychological functioning and social support perception can significantly increase the odds of experiencing NSSI among adolescents.

Therefore, further studies are needed for an in-depth analysis to understand these patients’ peculiar family characteristics. The present study aims to expand the knowledge on NSSI family interactive dynamics using a multimethod approach and a rigorous semi-standardized tool, the Lausanne Trilogue Play (LTP) [30]. The Lausanne Trilogue Play allows direct assessment of triadic family dynamics in four relational configurations through an observational procedure. The coding system enables the investigation of structural and dynamic aspects of triadic interactions, the “parent-parent-children unit”, which is composed by the co-parenting subsystem and the developmental subsystem. The child has an important role that implies that he must interact with his parents and provide them with enough cues to be appropriate in their initiatives and supervise them optimally. This role is manifested in the observation situation through behaviors that depend closely on his level of physical and psychological development, the LTP allows the observation of his interactive competencies that are directly active in the family play [30]. Several recent studies have shown the LTP procedure’s ability to identify the dysfunctional quality of family dynamics in clinical samples [31,32,33,34,35,36,37]. 

The present study aims to analyze and define the psychopathological and interactive-relational characteristics of subjects with NSSI through a multimethod approach and a case-control study. In line with the literature, we hypothesize that the non-suicidal self-injury adolescent patients show higher scores in both internalizing and externalizing disorders scales [10], in addition to difficulties in recognizing and describing their own feelings [14]. Concerning family interaction dynamics, it is possible to hypothesize that families with NSSI adolescents show a tense family climate [19], and a lower quality of family interactions compared to the control group [20,21]. We expected to observe, during family play, a significantly lower ability of emotional regulation in NSSI adolescents compared to the control group.

## 2. Materials and Methods

### 2.1. Participants and Procedure

Thirty-one patients with NSSI and thirty-one patients without NSSI paired for sex, age, and psychiatric diagnosis (ICD 10) were recruited consecutively among two Child Neuropsychiatry Units in Padua during the two-year period before the COVID-19 pandemic. 58 (93.55%) patients were female, and 4 (6.45%) were male; the age range was 13 to 16 years old (M = 14.9; SD = 1.2). All subjects had an ongoing psychopathological diagnosis according to the ICD-10. Out of 62 subjects, 27 showed psychopathological comorbidities. The matching of cases to controls was carried out according to the homogeneity criterion with regards to the main diagnostic category, gender, and age of subjects. Among the NSSI group, 12 subjects (39%) reported occasional self-harm episodes (<5/year). In contrast, 19 (61%) reported habitual self-harm behaviors, which fulfilled the DSM 5 diagnostic criteria for NSSI; that is, they reported more than 5 self-harm episodes in a year. As for sociodemographic characteristics, we considered the subject and parent age, gender, family civil status, and the number of siblings. As for the subjects’ clinical information, 24 controls and 24 case subjects had an ongoing diagnosis of emotional and mood disorders (F30–39, F40–48), 6 subjects in each group had an ongoing diagnosis of behavior and personality disorders (F60–69, F90–98), and only 1 subject in each group had an ongoing diagnosis of anorexia nervosa (F50–59). 48.4% (*n* = 15) case subjects and 38.7% (*n* = 12) controls showed comorbidity. 41.9% (*n* = 13) case subjects and 45% (*n* = 15) controls were undergoing pharmacological treatment.

As exclusion criteria, we applied: comorbidity of autism spectrum disorders, intellectual disability, and other medical conditions. 

Ten out of the families involved included divorced or separated parents. The parents’ age ranged from 35 to 60 years old for mothers (mean age for case subjects’ mothers = 46.3, SD = 6.61; mean age for controls’ mothers = 47.9, SD = 7.06), and between 33 and 71 years old for fathers (mean age for case subjects’ fathers = 49.7, SD = 8.02; mean age for controls’ fathers = 52.6, SD = 7.06). Overall, the homogeneity criteria between the two samples were fulfilled. 

The psychological assessment was carried out in 4 sessions according to the following protocol: a neuropsychiatrist and a psychologist held separate clinical interviews with the adolescent and their parents. During the psychological assessment, the adolescent completed the selected clinical questionnaires. The psychologist administered the Lausanne Trilogue Play (LTP) [30] during the second or third session.

After the psychological and neuropsychiatric evaluations, a final interview was held with the patients together with their parents and both the operators mentioned above to communicate the diagnosis and therapeutic recommendations. During the final interview, the clinical staff explained the aim of the research project to the families and obtained signed informed consent from both the young patients and their parents. They, therefore, agreed to the use of selected data for this study. The research project was approved by our Ethics Committee (Ethical-Committee approval CEP 204 SC).

### 2.2. Instruments

Achenbach Questionnaires [38]—Child Behaviour Checklist 6–18 (CBCL) parent-report; Youth Self Report 11–18 (YSR) self-report: the CBCL is a questionnaire (report form) designed for parents (referring to the last six months of their child’s life); the YSR is the children and adolescents’ version (self-report) of the questionnaire. Both questionnaires produced two different profiles: one concerning general abilities (activities, social functioning, school performance) and how well the adolescent performs in sports, hobbies, autonomy and socialization, and at school; the other concerning behavioral and emotional problems, which were classified as either “normal”, “borderline”, or “clinical” according to 8 specific syndrome scales. The syndrome scales refer to various psychopathological symptoms: anxiety/depression, withdrawal, somatization, social problems, thought-related problems, attention problems, and aggressive and rule-breaking behavior. The symptoms are grouped into three categories: internalizing problems (anxiety, depression, withdrawal, somatization); externalizing problems (aggressive and rule-breaking behavior); and other problems (social problems, thought-related problems, attention problems). The questionnaire also includes a DSM-oriented scale, which helps the clinician orient towards a diagnostic hypothesis in line with the DSM criteria. The values for Cronbach’s Alpha for the YSR scales ranging from 0.85 to 0.89, and for the CBCL scales from 0.87 to 0.93.

Toronto Alexithymia Scale (TAS-20) [39]: this is a 20-item self-reported questionnaire that measures the three factors defining alexithymia: “difficulty in identifying feelings”, “difficulty in communicating feelings to others”, and “externally-oriented thinking”. Respondents were classified as either non-alexithymic (score < 51), borderline (score 51–60), or alexithymic (score > 61). We used the validated Italian version of the TAS-20 with a Cronbach’s alpha coefficient ranging from 0.67 to 0.77. 

Lausanne Trilogue Play [30]: the LTP procedure is a semi-standardized play situation involving the two parents and the adolescent together. A setting specifically devised for the adolescents’ age group [34] involves the family planning either the adolescent’s birthday party or a field trip based on a four-part scenario based on the four possible triadic relational configurations: (1) 2 + 1, one parent plays with the adolescent while the other parent acts as an observer; (2) 2 + 1, the two parents swap roles; (3) 3, all three family members play together; (4) 2 + 1, the parents play together while the adolescent acts as an observer. The procedure is recorded using several video cameras pointed towards the family to obtain images appropriate for coding, based on the recommendations of the FAAS manual (Family Alliance Assessment Scale 6.3) [40,41] adapted for adolescents [34]. This coding system is based on the evaluation of the 15 variables forming the construct of Family Alliance: posture and gaze, inclusion of partners, role implication, structure, co-construction, parental scaffolding, family warmth, validation, authenticity, interactive mistakes during activities, interactive mistakes during transitions, support, conflicts, involvement, and self-regulation. Each scale provides an evaluation of a three-point scoring system, ranging from 1 (“inappropriate”) to 3 (“appropriate”) for each part, with a Cronbach’s alpha coefficient for this study ranging from 0.93 to 0.94. The scoring is carried out by two independent coders who received specific training in the LTP procedure and are not informed about the family’s situation; coders achieved an overall consistency calculated using Cohen’s kappa of 0.90. 

### 2.3. Data Analysis 

Data were analyzed using the software Jamovi 1.1.9 (2020). Descriptive statistics, like frequency and percentage for categorical variables, and mean and standard deviation for continuous variables, were reported. After an initial exploratory phase in which the descriptive statistics for each variable were investigated, we used parametric (Student’s *t*-test) statistical tests for hypothesis verification. To verify our hypotheses regarding frequency, a χ^2^ test was used. 

## 3. Results

### 3.1. NSSI Psychopathological Characteristics

Table 1 shows the descriptive and *t*-test analysis for each variable of the YRS and CBCL questionnaires for both study groups. For both groups and each scale, the recorded percentage frequencies of the clinical, borderline, and normative range scores and χ^2^ are reported in Table 2. 

In reason of a small sample, we have chosen to analyzing results with medium (d = 0.5) to large effect (d = 0.8). Student’s *t*-test for YSR show statistically significant differences between the two groups in the psychopathological profile. Specifically, what differentiates the non-suicidal self-injury group from the control group is a higher presence of internalizing problems related to anxiety and depression (*t*(57) = 2.687; *p* = 0.009; d = −0.70 95% CI [−1.24–0.15]), thought problems (*t*(57) = 3.222; *p* = 0.002 d = −0.84 95% CI [−1.39–0.27]), affective problems (*t*(57) = 3.083; *p* = 0.003; d = −0.80 95% CI [−1.35–0.24]), and social problems (*t*(57) = 2.719; *p* = 0.009; d = −0.71 95% CI [−1.25–0.16]).

χ^2^ test verifies the nonequivalence hypothesis between the two groups in the following scales: anxious/depressed (χ^2^ = 7.82; *p* = 0.020); thought problems (χ^2^ = 8.04; *p* = 0.018), and affective problems (χ^2^ = 8.32; *p* = 0.016).

Student’s *t*-test for CBCL shows statistically significant differences between the NSSI and the non-NSSI group in the following scales: internalizing problems (*t*(60) = 2.337, *p* = 0.023; d = −0.59 95% CI [−1.11–0.07]), externalizing problems (*t*(60) = 2.136, *p* = 0.037; d = −0.54 95% CI [−1.05–0.02), total problems (*t*(60) = 2.394; *p* = 0.020; d = −0.61 95% CI [−1.12–0.08]), anxious/depressed (*t*(60) = 2.187; *p* = 0.033; d = −0.55 95% CI [−1.07–0.03]), withdrawn/depressed (*t*(60) = 2.008; *p* = 0.049; d = −0.51 95% CI [−1.02–0.008), rule-breaking behavior (*t*(60) = 2.156, *p* = 0.035; d = −0.55 95% CI [−1.06–0.02]), affective problems (*t*(60) = 3.362; *p* = 0.001; d = −0.85 95% CI [−1.39–0.30]). The χ^2^ test shows statistically significant difference between the two groups in the following scales: thought problems (χ^2^ = 9.20; *p* = 0.010) and affective problems (χ^2^ = 10.7; *p* = 0.005). 

### 3.2. Alexithymia in NSSI Adolescents

The descriptive statistics relating to the total and the three scales in the two groups (NSSI and non-NSSI) are reported in Table 3. 

On average, the total scores fall within the “clinical” range (cut off > 60) for both groups with no statistically significant difference in χ^2^ test (χ^2^ = 2.88; *p* = 0.236). A statistically significant difference exists between the NSSI group and the non-NSSI group for the “difficulty identifying feelings” scale (*t*(59) = 3.40; *p* = 0.001; d = −0.87 95% CI [−1.41–0.31]). 

### 3.3. Quality of Family Interactions 

In order to investigate the differences between the interactive family dynamics in the two groups, we calculated the descriptive statistics referring to the global score in the Lausanne Trilogue Play (LTP) (Table 3). Please note that a higher score indicates higher family interaction quality, as observed by the procedure. 

In reason of a small sample, we have chosen to analyzing results with medium (d = 0.5) to large effect (d = 0.8). For all analyzed score, we observed lower mean scores in the case group compared to the controls. We found statistically significant differences between the two groups in the following LTP variables: inclusion of partners (*t*(55) = −2.185; *p* = 0.033; d = 0.58 95% CI [0.03–1.12]), child involvement (*t*(55) = −2.464; *p* = 0.017; d = 0.65 95% CI [0.09–1.20]), child self-regulation (*t*(55) = −2.366; *p* = 0.022; d = 0.63 95% CI [0.07–1.17]).

## 4. Discussion

The present study aims to analyze and define the psychopathological and family interactive-relational characteristics related to non-suicidal self-injury through a case-control study using a multi-method approach. A control group of adolescents without NSSI have been paired for sex, age, and psychiatric diagnosis. As for psychopathological characteristics, NSSI patients show higher levels of emotional-behavioral problems compared to controls with the same diagnosis and without episodes of NSSI behaviors. This result is consistent with our initial hypotheses. In particular, affective, anxious/depressive, withdrawal, thought, anxiety, and social problems have emerged, confirming a general presence of mostly an internalizing disorder in patients with NSSI. In the present study, the behavioral symptomatology was investigated through self and parent-report questionnaires. Both parents and children agree in observing higher levels of relational and internalizing problems associated with non-suicidal self-injury [10], which express an interpersonal and intrapersonal vulnerability of the NSSI subjects.

Moreover, data from the present study suggest that, for the same diagnosis, non-suicidal self-injury itself leads to significantly higher levels of severity for affective problems. In the NSSI subgroup, the parents of the NSSI patients also reported higher levels of attention and rule-breaking behavior problems than greater internalizing problems. Such findings could be an indicator of the egosyntonia with which the adolescents who self-harm experience specific dimensions of behavioral dysfunctionality, which presumably include self-aggressive behaviors.

Regarding the alexithymic functioning, our study confirms previous studies’ results, observing how alexithymia constitutes a typical trait of NSSI subjects [11,14,31]. Analyzing our results, it can be noticed how the “difficulty identifying feelings” scores are statistically higher for the NSSI group. Similar to what we observed for internalizing problems, the study suggests an association between alexithymia and NSSI. The results indicate the existence of a particular type of internalizing alexithymia and indicate that the specificity of self-harm lies primarily in the subjects’ difficulty in recognizing their own emotions rather than in a relational difficulty (communicating one’s emotions).

The present study also adds relevant and innovative data to the study of NSSI patients, inasmuch as it aimed to investigate the families’ interaction dynamics through the semi-standardized observational Lausanne Trilogue Play procedure. The results highlight a lower quality of the NSSI subjects’ family interactions in the “Inclusion of partners”, “Child Involvement”, and “Child self-regulation” variables. On a qualitative level, the comparison of the means shows a poorer quality of the NSSI group’s global interactive-relational functioning, in line with the literature showing an association between maladaptive family functioning and risk of engaging in NSSI behaviors [16,20,22,23,24,25,29]. To the best of our knowledge, our study represents the only case study carried out through a multi-method and multi-informant approach in the research on NSSI. Our results further confirm the findings from a previous study which, through the exploration of a single case report [33], revealed the existence of specific dysfunctional interactive features characterizing the NSSI subjects’ family relationships, namely, the adoption of self and other exclusion behaviors by the members of the family triad, low validation of the adolescent’s emotional experiences, emotional detachment, and lower family cohesion. Additional characteristics of the family’s interaction dynamics related to the child’s self-harm that emerge from the present study include those concerning the child’s involvement and self-regulation skills. In line with the literature, these results highlight the lack of negative emotion regulation [13] and cognitive control [42] strategies.

Simultaneously, emotion dysregulation affects the quality of family interactions [24,25], which are characterized by the NSSI subjects’ difficulties in the involvement and self-regulation dimensions.

This study presents some limitations that need to be taken into account when interpreting the results. First of all, the small sample size makes the results not generalizable; secondly, the composition of our sample underlines the higher prevalence of women engaging in NSSI behaviors found in the literature. A study of Washburn and colleagues [43] finds a preponderance of around 90% of female patients in line with of results. Two systematic reviews confirm the gender differences in the prevalence of non-suicidal self-injury [10,44]. Specifically, Bresin and Schoenleber [44] found a more considerable gender difference in NSSI in clinical versus college/community samples. One possible explanation is that males with NSSI are less likely to seek treatment from a neuropsychiatric service [44].

Our study represents one of few studies that deepen interactive-relational characteristics of NSSI adolescents before the pandemic period. As confirmed by recent studies, non-suicidal self-injury in adolescents has significantly increased after the outbreak of COVID-19 [45,46] and worse family and parent-children relationships appear strongly related as predisposing factors [47].

Another limitation was represented by the fact that no healthy sample was enrolled to constitute a further control group comparison; further studies with these comparation groups (clinical and non-clinical) could try to explain the gender differences.

## 5. Conclusions

In conclusion, for patients who receive a basic psychiatric diagnosis, self-harm behaviors are associated with higher severity of the psychopathological problems concerning the depression-anxiety-withdrawal areas, thought problems, social problems, and alexithymia levels (concerning difficulty in identifying feelings). Qualitatively, NSSI subjects’ family interactions are characterized by fewer inclusions of all family partners into NSSI management, a lower level of self-regulation, and inadequate involvement of the adolescent who had engaged in NSSI. These results carry significant implications for the clinical management and therapeutic care of non-suicidal self-injury patients, and further confirm the need for an in-depth investigation of internalizing problems, alexithymia, and quality of family interactions. The Lausanne Trilogue Play analysis method has once again proven to be a useful routine tool for assessing such aspects [34,35]. It can also be used in combination with video feedback techniques throughout the treatment.

## Figures and Tables

**Table 1:** Youth Self Report (YSR), Child Behavior Checklist (CBCL) Mean (M), standard deviation (SD) and Student’s t (*t*-test) in the two groups.

			YSR			CBCL		
			NSSI (*n* = 30)	NON-NSSI (*n* = 29)	Student’s *t*-Test	NSSI (*n* = 31)	NON-NSSI (*n* = 31)	Student’s *t*-Test
	Internalizing Problems	M (SD)	68.63 (13.21)	64.62 (12.05)	*t*(57) = 1.218; *p* = 0.228	71.19 (8.34)	66.13 (8.71)	*t*(60) = 2.337; *p* = 0.023
	Externalizing Problems	M (SD)	56.43 (11.35)	54.55 (13.85)	*t*(57) = 0.572; *p* = 0.570	62.81 (7.58)	58.32 (8.90)	*t*(60) = 2.136; *p* = 0.037
	Total Problems	M (SD)	65.52 (10.76)	61.10 (10.74)	*t*(56) = 1.563; *p* = 0.124	68.35 (5.78)	63.97 (8.40)	*t*(60) = 2.394; *p* = 0.020
Syndrome Scales	Anxious/Depressed	M (SD)	74.00 (11.93)	65.48 (12.42)	*t*(57) = 2.687; *p* = 0.009	71.84 (12.55)	65.74 (9.14)	*t*(60) = 2.187; *p* = 0.033
	Withdrawn/Depressed	M (SD)	69.40 (12.00)	65.93 (13.61)	*t*(57) = 1.039; *p* = 0.303	72.10 (13.42)	66.00 (10.28)	*t*(60) = 2.008; *p* = 0.049
	Somatic Complaints	M (SD)	65.47 (9.99)	60.66 (9.44)	*t*(57) = 1.900; *p* = 0.062	66.42 (7.80)	62.81 (7.52)	*t*(60) = 1.856; *p* = 0.068
	Social Problems	M (SD)	67.13 (7.57)	61.52 (8.29)	*t*(57) = 2.719; *p* = 0.009	66.90 (9.20)	64.03 (9.75)	*t*(60) = 1.193; *p* = 0.238
	Thought Problems	M (SD)	67.47 (10.65)	59.21 (8.93)	*t*(57) = 3.222; *p* = 0.002	67.35 (7.75)	61.90 (7.84)	*t*(60) = 2.753; *p* = 0.008
	Attention Problems	M (SD)	65.50 (8.32)	62.69 (11.74)	*t*(57) = 1.063; *p* = 0.292	65.06 (8.49)	62.58 (9.88)	*t*(60) = 1.061; *p* = 0.293
	Rule-Breaking Behavior	M (SD)	59.93 (9.73)	56.38 (8.00)	*t*(57) = 1.530; *p* = 0.132	61.19 (7.84)	57.35 (6.07)	*t*(60) = 2.156; *p* = 0.035
	Aggressive Behavior	M (SD)	61.07 (10.58)	57.79 (7.20)	*t*(57) = 1.385; *p* = 0.171	63.29 (6.13)	60.19 (9.16)	*t*(60) = 1.564; *p* = 0.123
DSM Oriented Scales	Affective Problems	M (SD)	73.80 (11.22)	65.10 (10.41)	*t*(57) = 3.083; *p* = 0.003	73.81 (8.63)	66.58 (8.29)	*t*(60) = 3.362; *p* = 0.001
	Anxiety Problems	M (SD)	65.70 (7.61)	62.07 (9.45)	*t*(57) = 1.628; *p* = 0.109	66.77 (7.12)	65.87 (7.27)	*t*(60) = 0.494; *p* = 0.623
	Somatic Problems	M (SD)	63.80 (10.00)	59.90 (9.47)	*t*(57) = 1.538; *p* = 0.129	64.65 (9.11)	61.60 (6.26)	*t*(59) = 1.517; *p* = 0.135
	Attention/Deficit	M (SD)	60.93 (9.07)	57.62 (6.88)	*t*(57) = 1.576; *p* = 0.120	60.13 (6.03)	58.97 (7.86)	*t*(60) = 0.653; *p* = 0.516
	Oppositional Defiant Problems	M (SD)	59.10 (7.91)	56.72 (7.59)	*t*(57) = 1.176; *p* = 0.244	61.26 (6.63)	58.52 (7.05)	*t*(60) = 1.578; *p* = 0.120
	Conduct Problems	M (SD)	57.90 (9.02)	57.21 (7.42)	*t*(57) = 0.322; *p* = 0.749	60.00 (8.07)	57.00 (7.42)	*t*(60) = 1.523; *p* = 0.133

**Table 2:** Youth Self Report (YSR), Child Behavior Checklist (CBCL) χ^2^ test with frequency of the Clinical, Borderline, and Normative results in the two groups.

		YSR						CBCL					
		NSSI (*n* = 30)			NON-NSSI (*n* = 29)			NSSI (*n* = 31)			NON-NSSI (*n* = 31)		
		%C	%B	%N	%C	%B	%N	%C	%B	%N	%C	%B	%N
	Internalizing Problems	73.3	13.3	13.3	58.6	13.8	27.6	87.1	3.2	9.7	74.2	6.5	19.4
		χ^2^ 1.96; *p* = 0.376						χ^2^ 1.65; *p* = 0.438					
	Externalizing Problems	26.7	16.7	56.7	24.1	17.2	58.6	51.6	22.6	25.8	32.3	16.1	51.6
		χ^2^ 0.05; *p* = 0.975						χ^2^ 4.38; *p* = 0.112					
	Total Problems	72.4	10.3	17.2	41.4	20.7	37.9	87.1	3.2	9.7	71.0	9.7	19.4
		χ^2^ 5.70; *p* = 0.058						χ^2^ 2.51; *p* = 0.285					
Syndrome Scales	Anxious/Depressed	63.3	20.0	16.7	31.0	20.7	48.3	45.2	29.0	25.8	38.7	29.0	32.3
		χ^2^ 7.82; *p* = 0.020						χ^2^ 0.38; *p* = 0.829					
	Withdrawn/Depressed	43.3	13.3	43.3	27.6	13.8	58.6	48.4	12.9	38.7	32.3	19.4	48.4
		χ^2^ 1.71; *p* = 0.426						χ^2^ 1.73; *p* = 0.420					
	Somatic Complaints	30.0	23.3	46.7	6.9	34.5	58.6	41.9	22.6	35.5	25.8	16.1	58.1
		χ^2^ 5.26; *p* = 0.072						χ^2^ 3.21; *p* = 0.201					
	Social Problems	33.3	23.3	43.3	17.2	20.7	62.1	29.0	32.3	38.7	19.4	19.4	61.3
		χ^2^ 2.53; *p* = 0.282						χ^2^ 3.18; *p* = 0.204					
	Thought Problems	30.0	26.7	43.3	10.3	10.3	79.3	48.4	16.1	35.5	12.9	29.0	58.1
		χ^2^ 8.04; *p* = 0.018						χ^2^ 9.20; *p* = 0.010					
	Attention Problems	23.3	23.3	53.3	20.7	13.8	65.5	22.6	32.3	45.2	19.4	9.7	71.0
		χ^2^ 1.14; *p* = 0.567						χ^2^ 5.62; *p* = 0.060					
	Rule-Breaking Behavior	20.0	3.3	76.7	6.9	13.8	79.3	22.6	16.1	61.3	3.2	12.9	83.9
		χ^2^ 3.78; *p* = 0.151						χ^2^ 5.70; *p* = 0.058					
	Aggressive Behavior	16.7	13.3	70.0	3.4	17.2	79.3	12.9	41.9	45.2	12.9	19.4	67.7
		χ^2^ 2.85; *p* =0.240						χ^2^ 3.98; *p* = 0.137					
DSM Oriented Scales	Affective Problems	63.3	23.3	13.3	31.0	24.1	44.8	71.0	22.6	6.5	32.3	35.5	32.3
		χ^2^ 8.32; *p* = 0.016						χ^2^ 10.7; *p* = 0.005					
	Anxiety Problems	33.3	20.0	46.7	37.9	6.9	55.2	41.9	6.5	51.6	48.4	16.1	35.5
		χ^2^ 2.16; *p* = 0.339						χ^2^ 2.35; *p* = 0.308					
	Somatic Problems	23.3	23.3	53.3	10.3	17.2	72.4	41.9	12.9	45.2	13.3	23.3	63.3
		χ^2^ 2.59; *p* = 0.274						χ^2^ 6.33; *p* = 0.042					
	Attention/Deficit	6.7	20.0	73.3	3.4	24.1	72.4	9.7	12.9	77.4	12.9	12.9	74.2
		χ^2^ 0.42; *p* = 0.812						χ^2^ 0.164; *p* = 0.921					
	Oppositional Defiant Problems	10.0	26.7	63.3	6.9	17.2	75.9	9.7	25.8	64.5	6.5	19.4	74.2
		χ^2^ 1.10; *p* = 0.578						χ^2^ 0.70; *p* = 0.706					
	Conduct Problems	16.7	6.7	76.7	6.9	13.8	79.3	12.9	22.6	64.5	6.5	19.4	74.2
		χ^2^ 1.94; *p* = 0.380						χ^2^ 0.95; *p* = 0.621					

**Table 3:** Toronto Alexithymia Scale (TAS-20), Lausanne Trilogue Play (LTP) Mean (M), standard deviation (SD) and Student’s t (*t*-test) in the two groups.

**TAS-20**	**Groups**			
		**NSSI (*n* = 31)**	**NON-NSSI** **(*n* = 30)**	**Student’s *t*-Test**
Total Score	M (SD)	65.87 (9.98)	61.50 (10.50)	*t*(59) = 1.667; *p* = 0.101
Difficulty Identifying Feeling	M (SD)	25.42 (4.82)	20.50 (6.39)	*t*(59) = 3.404; *p* = 0.001
Difficult Describing Feeling	M (SD)	18.03 (4.47)	17.60 (4.41)	*t*(59) = 0.380; *p* = 0.705
Externally-Oriented Thinking	M (SD)	22.42 (4.60)	24.27 (5.68)	*t*(59) = −1.399; *p* = 0.167
**LTP Variables**	**Groups**			
		**NSSI (*n* = 30)**	**NON-NSSI** **(*n* = 27)**	**Student’s *t*-Test**
Total Score	M (SD)	106.93 (26.41)	115.89 (23.25)	*t*(55) = −1.352; *p* = 0.182
Posture and gazes	M (SD)	7.10 (2.31)	7.26 (2.30)	*t*(55) = −0.261; *p* = 0.795
Inclusion of partners	M (SD)	8.17 (2.34)	9.48 (2.19)	*t*(55) = −2.185; *p* = 0.033
Role implication	M (SD)	8.27 (2.48)	8.67 (2.08)	*t*(55) = −0.657; *p* = 0.514
Structure	M (SD)	6.00 (2.24)	6.19 (2.10)	*t*(55) = −0.330; *p* = 0.743
Co-construction	M (SD)	6.37 (2.36)	7.04 (1.99)	*t*(55) = −1.154; *p* = 0.254
Parental scaffolding	M (SD)	6.60 (2.03)	7.22 (1.97)	*t*(55) = −1.173; *p* = 0.246
Support	M (SD)	7.80 (2.16)	8.85 (2.01)	*t*(55) = −1.897; *p* = 0.063
Conflicts	M (SD)	8.70 (2.47)	9.04 (2.08)	*t*(55) = −0.554; *p* = 0.582
Child Involvement	M (SD)	6.57 (2.24)	8.04 (2.26)	*t*(55) = −2.464; *p* = 0.017
Child Self-regulation	M (SD)	6.57 (2.62)	8.07 (2.13)	*t*(55) = −2.366; *p* = 0.022
Interactive mistakes during activities	M (SD)	6.50 (2.32)	6.52 (2.06)	*t*(55) = −0.032; *p* = 0.975
Interactive mistakes during transitions	M (SD)	7.90 (2.52)	7.85 (2.16)	*t*(55) = 0.077; *p* = 0.939
Family warmth	M (SD)	6.03 (2.25)	6.56 (2.53)	*t*(55) = −0.824; *p* = 0.413
Validation	M (SD)	6.10 (1.92)	6.93 (2.20)	*t*(55) = −1.514; *p* = 0.136
Authenticity	M (SD)	8.30 (2.88)	8.74 (2.71)	*t*(55) = −0.593; *p* = 0.555

## Data Availability

Restrictions apply to the availability of these data. Data was obtained from a broader project about family interactions, approved by Institutional Ethical Commission, and are available from the Principal Investigation (MG) on reasonable request.
